# Automated Breast Cancer Detection in Digital Mammograms of Various Densities via Deep Learning

**DOI:** 10.3390/jpm10040211

**Published:** 2020-11-06

**Authors:** Yong Joon Suh, Jaewon Jung, Bum-Joo Cho

**Affiliations:** 1 Department of Breast and Endocrine Surgery, Hallym University Sacred Heart Hospital, Anyang 14068, Korea; nicizm@hallym.or.kr; 2 Medical Artificial Intelligence Center, Hallym University Medical Center, Anyang 14068, Korea; deepmedical.jw@gmail.com; 3 Department of Ophthalmology, Hallym University Sacred Heart Hospital, Anyang 14068, Korea

**Keywords:** breast cancer, mammography, breast density, artificial intelligence, deep learning, convolutional neural network

## Abstract

Mammography plays an important role in screening breast cancer among females, and artificial intelligence has enabled the automated detection of diseases on medical images. This study aimed to develop a deep learning model detecting breast cancer in digital mammograms of various densities and to evaluate the model performance compared to previous studies. From 1501 subjects who underwent digital mammography between February 2007 and May 2015, craniocaudal and mediolateral view mammograms were included and concatenated for each breast, ultimately producing 3002 merged images. Two convolutional neural networks were trained to detect any malignant lesion on the merged images. The performances were tested using 301 merged images from 284 subjects and compared to a meta-analysis including 12 previous deep learning studies. The mean area under the receiver-operating characteristic curve (AUC) for detecting breast cancer in each merged mammogram was 0.952 ± 0.005 by DenseNet-169 and 0.954 ± 0.020 by EfficientNet-B5, respectively. The performance for malignancy detection decreased as breast density increased (density A, mean AUC = 0.984 vs. density D, mean AUC = 0.902 by DenseNet-169). When patients’ age was used as a covariate for malignancy detection, the performance showed little change (mean AUC, 0.953 ± 0.005). The mean sensitivity and specificity of the DenseNet-169 (87 and 88%, respectively) surpassed the mean values (81 and 82%, respectively) obtained in a meta-analysis. Deep learning would work efficiently in screening breast cancer in digital mammograms of various densities, which could be maximized in breasts with lower parenchyma density.

## 1. Introduction

Breast cancer is the most common cancer among women worldwide [1,2]. Over the last several decades, mammography has played an important role in breast cancer screening [3] and helped to reduce cancer-associated mortality rates [1]. Breast cancer is known to have an asymptomatic phase that can be detected by mammography only [4], and approximately 10% of patients undergoing mammography are reported to be recalled for further evaluation [5], among whom 8 to 10% needed a breast biopsy.

Of note, it requires the careful attention of a radiologist to read a mammogram to detect breast cancer, usually taking 30–60 s per each image [6]. Nevertheless, the sensitivity and specificity of mammogram reading by human radiologists have been reported as only 77–87% and 89–97%, respectively [7]. Recently, double reading is advocated in most screening programs, but this would worsen the time burden of human radiologists even more [4].

Recently, the progress of artificial intelligence (AI) has enabled automated disease detection on medical images in radiology, pathology, and even gastroenterology [8,9,10,11]. For breast cancer screening, several deep learning studies have also been performed, reporting sensitivities of 86.1–93.0% and specificities ranging from 79.0 to 90.0% [12,13,14,15]. Nevertheless, there are only few publications on AI-based breast cancer detection in mammograms among Asians who have higher breast densities compared to Caucasians. Breast density may affect the cancer detection rate in mammographic images [16,17,18,19,20].

Therefore, in this study, we aimed to develop and validate a deep learning model that automatically detects malignant breast lesions on digital mammograms of Asians, and investigated the performance of the model according to the grade of breast density. To maximize the performance of the model, we adopted a unique preprocessing method. Additionally, we endeavored to perform a meta-analysis for comparison using available studies on AI-based breast cancer detection. To our knowledge, this is one of the largest studies performed on Asians.

## 2. Methods

### 2.1. Study Subjects

Female patients who underwent digital mammography in the department of breast and endocrine surgery of Hallym University Sacred Heart Hospital between February 2007 and May 2015 were sequentially involved in this study. Only subjects 18 years of age or older and not having a history of previous breast surgery were included. Subjects without available medical records or pathological confirmation for a suspicious breast lesion, missing mammograms, or having poor-quality mammograms hindering proper interpretation due to noise, defocusing, or inadequate positioning were excluded. This study was approved by the Institutional Review Board (No. 2019-03-004) and adhered to the tenets of the declaration of Helsinki. The Institutional Review Board waived the requirement of written informed consent because this study involved no more than the minimal risk to subjects.

From the involved subjects, craniocaudal and mediolateral oblique view mammograms of each breast were retrieved using the picture archiving and communication system (PACS) of Hallym University Sacred Heart Hospital with a resolution of 2560 x 3328 pixels. Digital mammography protocols were in accordance with the European Federation of Organizations for Medical Physics. A Lorad Selenia digital mammography unit (Hologic Incorporated, Bedford, MA, USA) was used to capture all images. The performance of the unit was assured within a set and acceptable range implemented for quality control during the study period.

**Figure 1:**
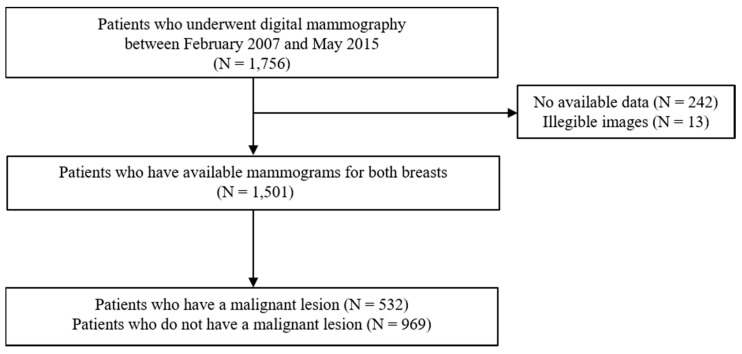
Flow diagram for the subject enrollment in this study.

Any personal information, annotation, or displayed information explaining laterality or type of mammography view was removed from the image. Then, all mammograms were re-reviewed and evaluated by two radiologists for the presence of any malignant lesion. Medical records, previous mammography readings, and pathological reports for any possibly malignant breast lesion were retrospectively investigated, following up for at least 5 years after the mammography examination. A malignant lesion had to be confirmed based on surgical histopathology. The participation flowchart is presented in Figure 1.

### 2.2. Data Preprocessing

Before analyses, all images were resized into the size of 1000 x 1300 pixels. Then, the images were preprocessed using a contrast limited adaptive histogram equalization (CLAHE) algorithm to minimize interimage contrast differences [21]. The CLAHE is a modification of an adaptive histogram equalization which is an image processing algorithm that transforms the brightness of each pixel in the image, applying histogram equalization locally in the neighboring pixel regions to enhance the local contrast of an image [22,23]. The CLAHE algorithm solves the noise overamplification problem of the adaptive histogram equalization, by limiting the cumulative density function slope when calculating histogram equalization [22,23]. In this study, the CLAHE was implemented using OpenCV library version 4.1.2.30 in Python programming language. All mammograms for left breasts were vertically flipped, making the images in a similar format to those of the right breasts, to make a unified deep learning model for right and left breasts.

Next, for each breast, craniocaudal and mediolateral oblique view images were cropped, removing marginal 10% of empty space, and were concatenated horizontally. Finally, the concatenated images were resized to the size of 900 x 650 pixels.

### 2.3. Dataset Construction

All the merged images were classified into two categories, malignant vs. benign, based on the presence or absence of malignant lesions in the images. The whole dataset was split into the training and testing datasets, allocating 10% of each category’s images randomly in the test dataset. Then, the resting training set was further divided into the proper training dataset and the tuning dataset at a ratio of 8:1, using random allocation by category. The training, tuning, and test datasets were mutually exclusive and collectively exhaustive.

Because the malignancy group was only approximately one-fifth of the non-malignant group in size, the malignancy group in the training dataset was augmented to mitigate the class imbalance. The images in the malignancy group of the training dataset were amplified as large as five times, producing the 10 and 20% magnified images and the 10 and 20% reduced images of the dataset.

### 2.4. Training Convolutional Neural Networks (CNNs)

To develop a deep learning model, two types of CNN architecture were used: DenseNet-169 and EfficientNet-B5. Briefly, DenseNet-169 is a CNN structure characterized by a dense block in which the input feature maps of each former sub-block are concatenated and then used as the input feature map of a certain sub-block [24]. This dense connectivity helps to resolve vanishing gradient problems and reduce the number of parameters [24]. EfficientNet-B5 was designed using a MBconv block, controlling the balance between the width, depth and resolution of a network at once via reinforcement learning [25]. This network outperformed previous networks on image classification using ImageNet dataset with fewer parameters and inference times [25]. These two CNN models were pretrained using the ImageNet dataset and were fine-tuned using the training dataset in this study.

An Adam optimizer was used for binary cross-entropy minimization using a beta1 of 0.9 and beta2 of 0.999. The initial learning rate was 10^−4^, and the learning rate was reduced by 10% every 10 epochs until when the rate reached 10^−7^. The batch size was set to 4, and the weight decay rate was 10^−4^. Early stopping was used after 30 epochs with a patience value of 20. Dropout was not used for DenseNet-169 but was used for EfficientNet-B5, with a dropout rate of 0.4. The Pytorch framework was used on the NVIDIA GeForce Titan RTX graphics processing unit.

### 2.5. Gradient-Weighted Class Activation Mapping (Grad-CAM)

Gradient-weighed class activation mapping (Grad-CAM) was used to show the region of interest recognized by the AI models [26]. Grad-CAM is a modified version of class activation mapping which requires replacing an existing fully connected layer with global average pooling (GAP) and a new fully connected layer, and re-training the network [27]. Grad-CAM works based on feature maps of an input image and its gradient [26]. The gradient is pooled via GAP, and the final color map is obtained through ReLU activation of the summation of multiplications of feature maps by the pooled gradient [26].

### 2.6. Meta-Analysis

Relevant articles were searched in the Pubmed, Embase, and Cochrane databases. The protocol was based on the Preferred Reporting Items for Systemic Reviews and Meta-Analysis (PRISMA) guideline. Authors, publication date, sample size, sensitivity, specificity, positive predictive value, and negative predictive value were extracted. Analysis was performed by RevMan 5.3 (Cochrane Collaboration, London, UK). A fixed-effects model was utilized in the presence of statistical homogeneity. However, a random-effects model was preferred if significant heterogeneity among the included studies was identified.

### 2.7. Statistical Analysis

After training, the performances of deep learning models were evaluated using the initially fixed testing dataset. The performances were evaluated three times using three different random seeds for the tuning dataset. The areas under receiver-operating characteristic (ROC) curves (AUCs) were calculated. The accuracy, sensitivity, specificity, positive predictive value, and negative predictive values were obtained on the ROC curves at the point maximizing Youden’s J statistic, or the sum of sensitivity and specificity minus one. Continuous variables were expressed as mean ± standard deviation, and a *p* value of <0.05 was considered statistically significant.

## 3. Results

### 3.1. Clinical Demographics of Subjects

Ultimately, a total of 3002 merged mammograms generated from 1501 patients were included in the study. The mean age of the participants was 48.9 ± 11.1 years. The whole dataset contained 537 malignant images and 2465 non-malignant images. The malignancy group was older than the non-malignancy group (52.7 ± 11.2 years old vs. 48.1 ± 10.9 years old; *p* <0.001). There were more images of dense breasts, density grade C or D (2256, 75.1%). Data composition of the training and testing datasets is presented in Table 1. The testing dataset contained 301 images including 54 images classified as malignant and 247 non-malignant images. The mean age of the participants who took the mammograms in the test dataset was 49.9 ± 10.9 years.

### 3.2. Performance of CNN Models for Breast Cancer Detection

**Figure 2:**
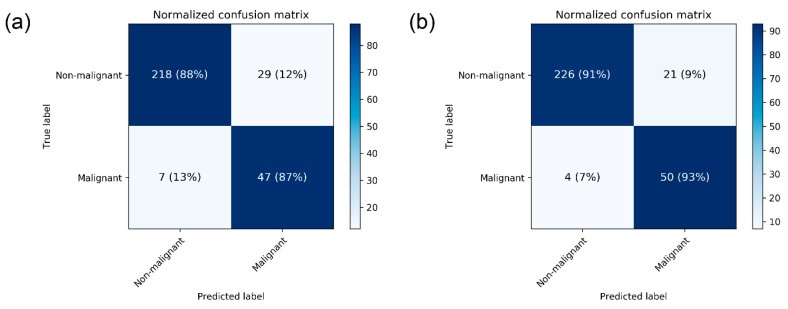
Heatmap of confusion matrix for breast cancer detection by the best performing (**a**) DenseNet-169 and (**b**) EfficientNet-B5.

The performance metrics of CNN models are presented in Table 2. The mean AUC for breast cancer detection in mammograms was 0.952 ± 0.005 by DenseNet-169 and 0.954 ± 0.020 by EfficientNet-B5. The mean accuracy was 88.1 ± 0.2% by DenseNet-169 and 87.9 ± 4.7 by EfficientNet-B5. For the DenseNet-169 model, mean sensitivity and specificity values were 87.0 ± 0.0 and 88.4 ± 0.2, respectively. The normalized confusion matrix for each CNN structure differentiating malignant images from non-malignant ones is presented in Figure 2.

### 3.3. Sub-Group Analyses

**Figure 3:**
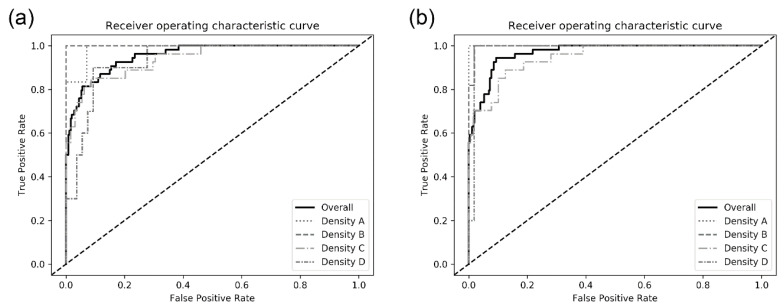
Receiver operating characteristic curves for detecting breast cancer on digital mammograms by the best performing (**a**) DenseNet-169 and (**b**) EfficientNet-B5.

The model performances among sub-groups by breast density in the test dataset are also presented in Table 2. There was an increasing tendency in the model performance as the breast density decreased. For the DenseNet-169 model, the mean AUC detecting malignancy in breasts with density A was higher than that in breasts with density D (0.984 ± 0.007 vs. 0.902 ± 0.033). The ROC curves of each CNN architecture for sub-groups are presented in Figure 3.

When patients’ age was additionally used in combination with mammogram for DenseNet-169, the overall performance of detecting breast cancer was not so improved (mean AUC, 0.953 ± 0.005). The mean AUC in the sub-group of density grade B reached 0.989 ± 0.009, but the AUCs in other sub-groups were nearly stationary. The performance metrics are presented in Supplementary Table S1.

### 3.4. Grad-CAM

**Figure 4:**
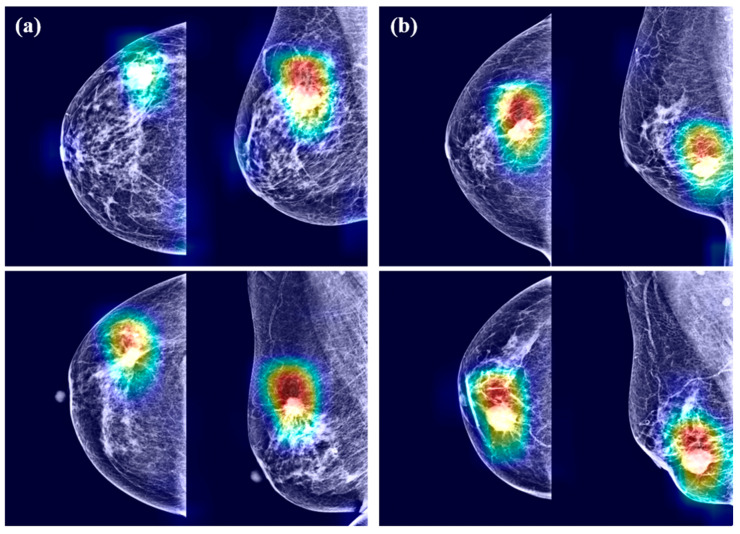
Gradient-weighed class activation mapping for mammograms having breast cancer by (**a**) DenseNet-169 and (**b**) EfficientNet-B5.

Examples of Grad-CAM images are presented in Figure 4. The CNN models detected malignant lesions efficiently. In the merged mammograms, the malignant lesions were mostly detected appropriately in both craniocaudal and mediolateral oblique view images. The CNN models focused on the interface between breast cancer and surrounding parenchyma. The radiopaque area contributed to cancer prediction more than the radiolucent area. Of note, the CNN models tended to identify the abnormalities in the form of mass or calcification rather than architectural distortion or asymmetry. However, radiopaque structures with normal structures were disregarded because they were likely to exist in most images. Grad-CAM also spanned areas beyond breast cancer, which means the importance of not only breast cancer itself but also surrounding parenchyma.

### 3.5. Meta-Analysis

In meta-analysis, 12 available deep learning studies were selected, where we could extract all necessary data for comparison [13,14,15,17,28,29,30,31,32,33,34,35]. A pooled analysis showed that sensitivity was 0.81 ± 0.01 and specificity was 0.82 ± 0.01 (Table 3). The present study showed better performances compared to the evaluated performances in our meta-analysis, although high heterogeneity was observed. The heterogeneity originated from different sample sizes, a discordant design, and unexplained variables.

## 4. Discussion

In the present study, we developed two CNN models for automatic breast cancer detection using digital mammograms collected originally from our institution. Two images per breast were concatenated and used for training by the DenseNet-169 and EfficientNet-B5 models. The mean AUC reached 0.952 ± 0.005 by DenseNet-169 and 0.954 ± 0.020 using EfficientNet-B5. The mean AUC was increased in sub-groups involving breasts with lower parenchyma density.

Previously, mean AUCs in similar studies have ranged from 0.70 to 0.96 [3,5,28,36,37,38,39]. The wide range of AUC values comes from using heterogeneous data or a small amount of data. Additionally, the bias of different mammography equipment manufacturers may contribute to AUC variability, because there is the difference in vendor-specific contrast/brightness characteristics [29]. Nonetheless, deep learning applied to mammography can provide automated assistance in breast cancer detection. When we performed the meta-analysis, a pooled analysis showed that the sensitivity was 0.81 ± 0.01 and specificity was 0.82 ± 0.01. The present study showed a good performance and general agreement with the previous studies.

The majority of cancer cases that were initially undetected in screening mammograms correlate with dense breast tissue (density equal to C or D) [40]. Large numbers of breast lesions are occluded by overlapping fibroglandular tissues in two-dimensional images. Our results showed high performance even in patients with a dense breast tissue, grade C or D, although there a higher performance was seen in patients with grade A or B. Researchers already reported the lower detection rates in dense breast, where masking could occur [29,39,40]. Our models can assist radiologists in mammogram interpretation resulting in higher accuracy and detection rates. Assisting and improving human performance in the medical field is one of the roles anticipated to be undertaken by artificial intelligence.

Because breast density is higher in Asians, the value of mammography would be reduced among Asians compared to Westerners. Although there is certain evidence that high tissue density causes breast cancer, a higher density can interfere with mammograms, which has the potential for lowering the detection rate [16,17,18,19,20]. This might mean mammograms with grade C or D densities need automated support to assist the human eye in interpretation. Thus, these algorithms could be used for detecting breast cancer in Asians because our algorithms were not influenced by breast density.

The Breast Imaging-Reporting and Data System (BI-RADS) categories consider calcification, mass, architectural distortion, and associated findings to homogenize the data collection and quality of mammography reports. However, these parameters sometimes intermingle in a complex way, which produces greater inter- and intraobserver variability [41]. Our study focused on disease discrimination not BI-RADS categories. Therefore, our current algorithms do not fulfill the expectation that BI-RADS would reduce variability in mammogram interpretation. Because BI-RADS categories work for disease discrimination, our current algorithms will still help radiologists to interpret mammography.

Downscaling, downsampling, or focusing on only a small region of interest hampers artificial intelligence performance as digital mammography screening relies on fine details. It is important to visualize the whole breast to assess architectural distortion. Machine learning in mammography should not only deal with high-resolution images but also considers the standard four views concurrently. Breast asymmetry is important for breast cancer detection because it is known that both breasts from the same patient tend to have a high degree of symmetry [42]. However, we merged two different views for each breast into one image. Thus, our models tended to find the abnormalities in the form of mass or calcification rather than architectural distortion or asymmetry. Considering these vulnerable points, our models will be consistently upgraded for the detection of malignant breast lesions.

CAM uses combined pixels identified by the algorithm to be of interest and overlays a color-coded distribution on the image. The Grad-CAM shows highlighted areas representing regions, which were positive in predicting breast cancer, with red indicating areas of strong emphasis. The color distribution spans to a blue area, indicating little value. When algorithms make classification decisions, CAM illustrates regions where important features are extracted. Using this approach, radiologists can recognize the value of machine learning. This kind of visualization enables better communion with humans while retaining prediction accuracy. CAM will contribute to a wider adoption of machine learning techniques.

The present study has limitations. The number of patients sampled was small for machine learning analysis. However, our mammograms were strictly classified based on pathology confirmation and a 5-year follow-up period used to minimize the interval cancer risk. Second, the original images were derived from a single tertiary academic institution, to which more severe patients were referred from secondary institutions. Lastly, mammograms were generated using a single equipment vendor. Further research is needed to validate our model across institutions and vendors before it can be broadly implemented. Additionally, our research suffers from the usual limitations of observational studies.

## 5. Conclusions

Our deep learning models would help to interpret digital mammography to identify patients with breast cancer. Using this strategy, the burden on radiologists could be reduced considerably.

## Figures and Tables

**Table 1:** Data composition for digital mammograms in the training and testing datasets.

		Whole Dataset		Training Set		Test Set	
		Breast *n*	Patient *n*	Breast *n*	Patient *n*	Breast *n*	Patient *n*
Overall		3002	1501	2701	1484	301	284
Non-malignant		2465	1496	2218	1427	247	235
Malignant		537	532	483	478	54	54
Breast density	A	152	76	132	74	20	18
	B	594	297	532	292	62	57
	C	1560	780	1405	774	155	149
	D	696	348	632	344	64	60

**Table 2:** Performances of deep learning models for breast cancer detection in mammograms by breast density.

Breast Density/Model	Accuracy (%)	Sensitivity (%)	Specificity (%)	PPV (%)	NPV (%)	AUC
**Overall**						
DenseNet-169	88.1 ± 0.2	87.0 ± 0.0	88.4 ± 0.2	62.1 ± 0.5	96.9 ± 0.0	0.952 ± 0.005
EfficientNet-B5	87.9 ± 4.7	88.3 ± 4.7	87.9 ± 4.7	62.1 ± 9.9	97.2 ± 1.3	0.954 ± 0.020
**Density A**						
DenseNet-169	95.0 ± 0.0	100 ± 0.0	92.9 ± 0.0	85.7 ± 0.0	100.0 ± 0.0	0.984 ± 0.007
EfficientNet-B5	96.7 ± 2.9	100.0 ± 0.0	95.3 ± 4.1	90.5 ± 8.3	100.0 ± 0.0	0.988 ± 0.012
**Density B**						
DenseNet-169	96.2 ± 4.1	97.0 ± 5.3	96.1 ± 3.9	85.3 ± 14.3	99.3 ± 1.2	0.962 ± 0.041
EfficientNet-B5	95.2 ± 4.3	97.0 ± 5.3	94.8 ± 4.1	81.0 ± 12.9	99.3 ± 1.2	0.990 ± 0.009
**Density C**						
DenseNet-169	86.4 ± 6.2	87.7 ± 4.3	86.2 ± 6.7	58.8 ± 13.5	97.0 ± 1.1	0.950 ± 0.014
EfficientNet-B5	81.9 ± 5.1	84.0 ± 5.7	81.5 ± 5.2	49.6 ± 9.1	96.0 ± 1.6	0.940 ± 0.016
**Density D**						
DenseNet-169	84.3 ± 5.4	83.3 ± 5.8	84.6 ± 5.3	51.0 ± 11.5	96.5 ± 1.3	0.902 ± 0.033
EfficientNet-B5	85.9 ± 10.9	86.7 ± 11.5	85.8 ± 10.8	58.4 ± 28.2	97.1 ± 2.5	0.925 ± 0.055

PPV, positive predictive value; NPV, negative predictive value; AUC, area under the receiver operating characteristic curve.

**Table 3:** Forest plot of the previous studies showing the pooled (**a**) sensitivity and (**b**) specificity on performance of deep learning algorithm for breast cancer detection in mammograms. CI, confidence interval.

(a) Sensitivity			
		Sensitivity (95% CI)	
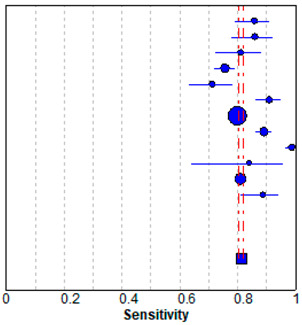	Regab (2019)	0.86	(0.79–0.91)
	Rodriguez–Ruiz (2019)	0.86	(0.78–0.92)
	Gastounioti (2018)	0.81	(0.72–0.88)
	Kim (2018)	0.76	(0.72–0.79)
	Becker (2017)	0.71	(0.63–0.79)
	Teare (2017)	0.91	(0.86–0.95)
	Akselrob-Ballin (2019)	0.80	(0.79–0.81)
	Cai (2019)	0.89	(0.86–0.92)
	Al-Masni (2018)	0.99	(0.96–1.00)
	Casti (2017)	0.84	(0.64–0.95)
	Sun (2017)	0.81	(0.79–0.83)
	Wang (2016)	0.89	(0.81–0.94)
	Pooled sensitivity = 0.81 (0.80–0.82)		
	I^2^ = 0.927		
(**b**) Specificity			
		Specificity (95% CI)	
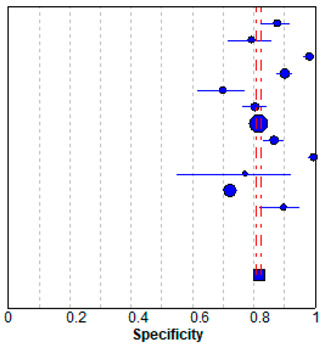	Regab (2019)	0.88	(0.82–0.92)
	Rodriguez–Ruiz (2019)	0.79	(0.72–0.86)
	Gastounioti (2018)	0.98	(0.96–0.99)
	Kim (2018)	0.90	(0.88–0.92)
	Becker (2017)	0.70	(0.62–0.77)
	Teare (2017)	0.80	(0.76–0.84)
	Akselrob-Ballin (2019)	0.82	(0.80–0.83)
	Cai (2019)	0.87	(0.83–0.90)
	Al-Masni (2018)	1.00	(0.98–1.00)
	Casti (2017)	0.77	(0.55–0.92)
	Sun (2017)	0.72	(0.70–0.74)
	Wang (2016)	0.90	(0.82–0.95)
	Pooled specificity = 0.82 (0.81–0.82)		
	I^2^ = 0.967		

## Supplementary Materials

The following are available online at https://www.mdpi.com/2075-4426/10/4/211/s1, Table S1: Performance for breast cancer detection in mammograms by the DenseNet-169 model using age as a covariate.

## References

[1] Harbeck N., Gnant M. (2017). Breast cancer. Lancet.

[2] Kelder A., Lederman D., Zheng B., Zigel Y. (2018). A new computer-aided detection approach based on analysis of local and global mammographic feature asymmetry. Med. Phys..

[3] Gardezi S.J.S., Elazab A., Lei B., Wang T. (2019). Breast Cancer Detection and Diagnosis Using Mammographic Data: Systematic Review. J. Med. Internet Res..

[4] Arevalo J., Gonzalez F.A., Ramos-Pollan R., Oliveira J.L., Guevara Lopez M.A. (2016). Representation learning for mammography mass lesion classification with convolutional neural networks. Comput. Methods Programs Biomed..

[5] Hamidinekoo A., Denton E., Rampun A., Honnor K., Zwiggelaar R. (2018). Deep learning in mammography and breast histology, an overview and future trends. Med. Image Anal..

[6] Le E.P.V., Wang Y., Huang Y., Hickman S., Gilbert F.J. (2019). Artificial intelligence in breast imaging. Clin. Radiol..

[7] Ribli D., Horvath A., Unger Z., Pollner P., Csabai I. (2018). Detecting and classifying lesions in mammograms with Deep Learning. Sci. Rep..

[8] Saba L., Biswas M., Kuppili V., Cuadrado Godia E., Suri H.S., Edla D.R., Omerzu T., Laird J.R., Khanna N.N., Mavrogeni S. (2019). The present and future of deep learning in radiology. Eur. J. Radiol..

[9] Niazi M.K.K., Parwani A.V., Gurcan M.N. (2019). Digital pathology and artificial intelligence. Lancet Oncol..

[10] Cho B.J., Bang C.S., Park S.W., Yang Y.J., Seo S.I., Lim H., Shin W.G., Hong J.T., Yoo Y.T., Hong S.H. (2019). Automated classification of gastric neoplasms in endoscopic images using a convolutional neural network. Endoscopy.

[11] Yang Y.J., Cho B.J., Lee M.J., Kim J.H., Lim H., Bang C.S., Jeong H.M., Hong J.T., Baik G.H. (2020). Automated Classification of Colorectal Neoplasms in White-Light Colonoscopy Images via Deep Learning. J. Clin. Med..

[12] Shen L., Margolies L.R., Rothstein J.H., Fluder E., McBride R., Sieh W. (2019). Deep Learning to Improve Breast Cancer Detection on Screening Mammography. Sci. Rep..

[13] Rodriguez-Ruiz A., Krupinski E., Mordang J.J., Schilling K., Heywang-Kobrunner S.H., Sechopoulos I., Mann R.M. (2019). Detection of Breast Cancer with Mammography: Effect of an Artificial Intelligence Support System. Radiology.

[14] Ragab D.A., Sharkas M., Marshall S., Ren J. (2019). Breast cancer detection using deep convolutional neural networks and support vector machines. PeerJ.

[15] Wang J., Yang X., Cai H., Tan W., Jin C., Li L. (2016). Discrimination of Breast Cancer with Microcalcifications on Mammography by Deep Learning. Sci. Rep..

[16] Boyd N.F., Guo H., Martin L.J., Sun L., Stone J., Fishell E., Jong R.A., Hislop G., Chiarelli A., Minkin S. (2007). Mammographic density and the risk and detection of breast cancer. N. Engl. J. Med..

[17] Al-Masni M.A., Al-Antari M.A., Park J.M., Gi G., Kim T.Y., Rivera P., Valarezo E., Choi M.T., Han S.M., Kim T.S. (2018). Simultaneous detection and classification of breast masses in digital mammograms via a deep learning YOLO-based CAD system. Comput. Methods Programs Biomed..

[18] Rajaram N., Mariapun S., Eriksson M., Tapia J., Kwan P.Y., Ho W.K., Harun F., Rahmat K., Czene K., Taib N.A. (2017). Differences in mammographic density between Asian and Caucasian populations: A comparative analysis. Breast Cancer Res. Treat..

[19] Freer P.E. (2015). Mammographic breast density: Impact on breast cancer risk and implications for screening. Radiographics.

[20] Brentnall A.R., Cuzick J., Buist D.S.M., Bowles E.J.A. (2018). Long-term Accuracy of Breast Cancer Risk Assessment Combining Classic Risk Factors and Breast Density. JAMA Oncol..

[21] Pizer S.M., Johnston R.E., Ericksen J.P., Yankaskas B.C., Muller K.E. (1990). Contrast-Limited Adaptive Histogram Equalization: Speed and Effectiveness. Proc. First Conf. Vis. Biomed. Comput..

[22] Pizer S.M., Amburn E.P., Austin J.D., Robert C., Geselowitz A., Greer T., Romeny B.T.H., Zimmerman J.B., Zuiderveld K. (1987). Adaptive Histogram Equalization and Its Variations. Comput. Vis. Graph. Image Process..

[23] Ketcham D.J., Lowe R., Weber W. (1976). Real-Time Image Enhancement Techniques. Semin. Image Process..

[24] Huang G., Liu Z., van der Maaten L., Weinberger K.Q. (2016). Densely Connected Convolutional Networks. arXiv.

[25] Tan M., Le Q.V. (2019). EfficientNet: Rethinking Model Scaling for Convolutional Neural Networks. arXiv.

[26] Selvaraju R.R., Cogswell M., Das A., Vedantam R., Parikh D., Batra D. (2016). Grad-CAM: Visual Explanations from Deep Networks via Gradient-based Localization. arXiv.

[27] Zhou B., Khosla A., Lapedriza A., Oliva A., Torralba A. (2015). Learning Deep Features for Discriminative Localization. arXiv.

[28] Gastounioti A., Oustimov A., Hsieh M.K., Pantalone L., Conant E.F., Kontos D. (2018). Using Convolutional Neural Networks for Enhanced Capture of Breast Parenchymal Complexity Patterns Associated with Breast Cancer Risk. Acad. Radiol..

[29] Kim E.K., Kim H.E., Han K., Kang B.J., Sohn Y.M., Woo O.H., Lee C.W. (2018). Applying Data-driven Imaging Biomarker in Mammography for Breast Cancer Screening: Preliminary Study. Sci. Rep..

[30] Becker A.S., Marcon M., Ghafoor S., Wurnig M.C., Frauenfelder T., Boss A. (2017). Deep Learning in Mammography: Diagnostic Accuracy of a Multipurpose Image Analysis Software in the Detection of Breast Cancer. Investig. Radiol..

[31] Teare P., Fishman M., Benzaquen O., Toledano E., Elnekave E. (2017). Malignancy Detection on Mammography Using Dual Deep Convolutional Neural Networks and Genetically Discovered False Color Input Enhancement. J. Digit. Imaging.

[32] Akselrod-Ballin A., Chorev M., Shoshan Y., Spiro A., Hazan A., Melamed R., Barkan E., Herzel E., Naor S., Karavani E. (2019). Predicting Breast Cancer by Applying Deep Learning to Linked Health Records and Mammograms. Radiology.

[33] Cai H., Huang Q., Rong W., Song Y., Li J., Wang J., Chen J., Li L. (2019). Breast Microcalcification Diagnosis Using Deep Convolutional Neural Network from Digital Mammograms. Comput. Math. Methods Med..

[34] Casti P., Mencattini A., Salmeri M., Ancona A., Lorusso M., Pepe M.L., Natale C.D., Martinelli E. (2017). Towards localization of malignant sites of asymmetry across bilateral mammograms. Comput. Methods Programs Biomed..

[35] Sun W., Tseng T.B., Zhang J., Qian W. (2017). Enhancing deep convolutional neural network scheme for breast cancer diagnosis with unlabeled data. Comput. Med. Imaging Graph..

[36] Yassin N.I.R., Omran S., El Houby E.M.F., Allam H. (2018). Machine learning techniques for breast cancer computer aided diagnosis using different image modalities: A systematic review. Comput. Methods Programs Biomed..

[37] Lee R.S., Gimenez F., Hoogi A., Miyake K.K., Gorovoy M., Rubin D.L. (2017). A curated mammography data set for use in computer-aided detection and diagnosis research. Sci. Data.

[38] Kooi T., Litjens G., van Ginneken B., Gubern-Merida A., Sanchez C.I., Mann R., den Heeten A., Karssemeijer N. (2017). Large scale deep learning for computer aided detection of mammographic lesions. Med. Image Anal..

[39] Yala A., Lehman C., Schuster T., Portnoi T., Barzilay R. (2019). A Deep Learning Mammography-based Model for Improved Breast Cancer Risk Prediction. Radiology.

[40] Garcia-Manso A., Garcia-Orellana C.J., Gonzalez-Velasco H.M., Gallardo-Caballero R., Macias-Macias M. (2013). Study of the effect of breast tissue density on detection of masses in mammograms. Comput. Math. Methods Med..

[41] Balleyguier C., Ayadi S., Van Nguyen K., Vanel D., Dromain C., Sigal R. (2007). BIRADS classification in mammography. Eur. J. Radiol..

[42] Bandeira Diniz J.O., Bandeira Diniz P.H., Azevedo Valente T.L., Correa Silva A., de Paiva A.C., Gattass M. (2018). Detection of mass regions in mammograms by bilateral analysis adapted to breast density using similarity indexes and convolutional neural networks. Comput. Methods Programs Biomed..

