# Supplementary material for: Automated Breast Cancer Detection in Digital Mammograms of Various Densities via Deep Learning
Source: J Pers Med. 2020 Nov 6;10(4):211. doi: 10.3390/jpm10040211 (PMC7711783; doi:10.3390/jpm10040211)
Supplement: Supplementary file 1 [file jpm-10-00211-s001.pdf]

Supplementary Table 1. Performance for breast cancer detection in mammograms by the DenseNet-169 model using age as a covariate

Breast density	Accuracy (%)	Sensitivity (%)	Specificity (%)	PPV (%)	NPV (%)	AUC
Overall	86.9 ± 1.1	86.4 ± 1.0	87.0 ± 1.1	59.4 ± 2.3	96.7 ± 0.3	0.953 ± 0.005
Density A	95.0 ± 0.0	100± 0.0	92.9 ± 0.0	85.7 ± 0.0	100.0 ± 0.0	0.976 ± 0.012
Density B	93.0 ± 6.1	90.9 ± 9.1	93.5 ± 5.7	77.0 ± 20.0	97.9 ± 2.1	0.989 ± 0.009
Density C	85.8 ± 5.7	87.7 ± 2.1	85.4 ± 6.5	57.3 ± 11.1	97.0 ± 0.7	0.953 ± 0.014
Density D	82.3 ± 8.6	80.0 ± 10.0	82.7 ± 8.3	48.2 ± 15.5	95.6 ± 2.5	0.899 ± 0.036
